# A Tissue Retrieval and Postharvest Processing Regimen for Rodent Reproductive Tissues Compatible with Long-Term Storage on the International Space Station and Postflight Biospecimen Sharing Program

**DOI:** 10.1155/2015/475935

**Published:** 2015-01-13

**Authors:** Vijayalaxmi Gupta, Lesya Holets-Bondar, Katherine F. Roby, George Enders, Joseph S. Tash

**Affiliations:** ^1^Department of Molecular & Integrative Physiology, University of Kansas Medical Center, Mail Stop 3050, 3901 Rainbow Boulevard, HLSIC 3098, Kansas City, KS 66160, USA; ^2^Department of Anatomy and Cell Biology, University of Kansas Medical Center, Kansas City, KS 66160, USA; ^3^Institute for Reproductive Health and Regenerative Medicine, University of Kansas Medical Center, Kansas City, KS 66160, USA

## Abstract

Collection and processing of tissues to preserve space flight effects from animals after return to Earth is challenging. Specimens must be harvested with minimal time after landing to minimize postflight readaptation alterations in protein expression/translation, posttranslational modifications, and expression, as well as changes in gene expression and tissue histological degradation after euthanasia. We report the development of a widely applicable strategy for determining the window of optimal species-specific and tissue-specific posteuthanasia harvest that can be utilized to integrate into multi-investigator Biospecimen Sharing Programs. We also determined methods for ISS-compatible long-term tissue storage (10 months at −80°C) that yield recovery of high quality mRNA and protein for western analysis after sample return. Our focus was reproductive tissues. The time following euthanasia where tissues could be collected and histological integrity was maintained varied with tissue and species ranging between 1 and 3 hours. RNA quality was preserved in key reproductive tissues fixed in RNA*later* up to 40 min after euthanasia. Postfixation processing was also standardized for safe shipment back to our laboratory. Our strategy can be adapted for other tissues under NASA's Biospecimen Sharing Program or similar multi-investigator tissue sharing opportunities.

## 1. Introduction

With the current paucity of opportunities for studying whole animal mammalian physiology in space flight, the Biospecimen Sharing Program (BSP) for postflight tissue collection offers the opportunity to broaden access to biological samples shortly after return and maximize the data generated from flight animal payloads. The logistics of space flight experiments involving live animals often requires harvesting tissues at a remote site, followed by shipping the specimens to the Principle Investigators' laboratories for detailed analysis. Furthermore, as the capabilities to house rodent and other animals on the International Space station (ISS), and to conduct long-term space flight experiments using animals are enabled, the need to harvest and fix tissues for long-term storage on ISS that will retain high quality RNA and protein for subsequent analysis in laboratories on Earth is also required. These approaches to live animal experimentation in space flight that include tissue harvest for multiple investigators require determination of (1) tissue-specific windows of time between euthanasia and tissue fixation that retain quality of histology and (2) tissue-specific windows of time for tissue fixation that retain high quality protein and RNA for subsequent analysis. Determination of these windows provides a quantitative, logical approach to generate appropriately prioritized and optimized tissue harvest and fixation logistics in a multi-investigator Biospecimen Sharing Program scenario, be it on Earth or during tissue harvest on the ISS. In addition, tissue storage methods should retain high sample quality under long-term storage, as samples may be harvested and stored on the ISS but may not be returned to Earth for many months, depending on ISS to Earth flight frequency and payload capacities. This issue has been addressed for preserving plant material for gene expression analysis [1], but there is no data available for animal tissues. Our participation in the BSP program involved tissue harvest from female mice at Kennedy Space Center, Florida, USA (KSC) for animals flown for 12–15 days in orbit on three space shuttle flights: STS-131, STS-133, and STS-135. In addition, we harvested tissues from male mice at the Institute for Biomedical Problems (IMBP) laboratory in Moscow, RU for animals flown for 30 days in orbit on the BION M1 satellite. Both the STS and BION series of flight experiments involved age- and time-matched ground control groups of animals. During the early flight planning phase of BION, there were possibilities that male or female mice would be flown and that male gerbil tissues may also be provided to the BSP. Thus, to be prepared for any of these possibilities, we undertook to determine the optimal tissue harvest windows for all of the species and reproductive tissues that we might be able to obtain. Our participation in these multi-investigator specimen sharing efforts covering four different primary flight PI experimental designs and harvest logistics necessitated a determination of the window of time between euthanasia and harvest and preservation of our tissues of interest that would allow us sufficient flexibility to obtain the highest possible quality of tissue for histopathology, RNA for gene-transcription analysis, and protein for expression and posttranslational modification analysis. A determination of these time windows is essential, since the tissues that are made available to the BSP investigators are provided after the primary flight PI's have obtained their tissues. Knowledge of the optimal windows for all of the tissues of interest aids in the preparation of targeted tissue harvest flow logistics that can provide each of the BSP team members the highest possible quality tissue, respectively. Therefore, as we report here, we developed a strategy to determine the window of time between euthanasia and fixation for retention of high quality histology for male and female reproductive organs. We also determined methods for long-term tissue storage for 10 months that provide for recovery of both high quality protein and RNA. These strategies are adaptable and can be applied to harvest and storage of other time-sensitive labile tissues from animals and plants. Furthermore, these methods can be used to optimize logistics and data collection under multi-investigator tissue harvest and sharing programs operated by any space agency, commercial entity, or flight platform.

## 2. Materials and Methods

### 2.1. Animals

Approximately 8 wk old male and female C57Bl/6J (Jackson Lab, Bar Harbor, ME) and ~10-month old male and female Mongolian gerbils (Charles River, Wilmington, MA) were used throughout this study. All animal use protocols were approved by the University of Kansas Institutional Animal Care and Use Committee (IACUC). Animals were maintained in standard cages with 12 : 12 h dark : light cycle, and standard food and water were provided ad libitum. All animals were euthanized using CO_2_ asphyxiation followed by cervical dislocation prior to tissue harvest.

### 2.2. Determination of the Limit of Time and Temperature between Euthanasia and Tissue Fixation

#### 2.2.1. Male Mice

Our protocol consisted of three groups of six mice each, with one mouse for each time point. In group A, all mice were euthanized at one time and the testes and epididymides were harvested and separated and then immediately placed in Ham's F-10 medium (Sigma Aldrich, St. Louis, MO)* on ice*. At time intervals of 0, 0.5, 1.0, 1.5, 2.0, and 2.5 hr each testis (one mouse per time point) and epididymis (2 mice per time point) were separated and then transferred from Ham's F-10 medium to Bouin's fixative (Sigma Aldrich, St. Louis, MO) at room temperature (RT; 21°C). In group B, the same procedure was followed, except that the testes and epididymides, separated, were placed in Ham's F-10 medium* at RT* until transfer to Bouin's at the same time interval as above. In group C, all mice were euthanized at one time, the* carcasses were maintained at (RT)*, and then at time 0, 0.5, 1.0, 1.5, 2.0, and 2.5 hr, the testis and epididymis were harvested from the carcasses, respectively. At each of the time points above, group C testes were placed in Bouin's solution and processed as per standard histology protocols, as detailed below. For all groups, the tissues fixed in Bouin's were processed as detailed in Section 2.3, below. At the same time point, group C epididymides were processed to obtain cauda sperm to assess sperm motility by computer assisted sperm analysis (CASA) [2]. For animals used for collection of cauda epididymal sperm for motility analysis, two animals were used at each time point.

#### 2.2.2. Female Mice

Female mice were euthanized (carcasses maintained at RT as per group C, above) and their ovaries and uteri were harvested at 0, 0.5, 1.0, 1.5, 2.0, 2.5, and 3.0 hr after euthanasia (one animal per time point). At the times indicated, the tissues were fixed at RT in Bouin's overnight and then processed for histology as detailed below (Section 2.3).

#### 2.2.3. Mongolian Gerbils

The six male gerbils were euthanized at one time, and the carcasses were kept at room temperature. At 0, 0.5, 1.0, 1.5, 2.0, and 3.0 hr after euthanasia, testis and epididymis were harvested from the carcasses and separated, respectively (one animal per time point). Similarly, all six female gerbils were euthanized and the carcasses were kept at RT. Ovaries and uterine horns were harvested from each carcass at the same time interval as the males. At the times indicated above, the tissues were placed in Bouin's fixative at RT and then processed for histology, as detailed below (Section 2.3). Sperm were immediately harvested from cauda epididymis as mentioned above, and sperm motility analysis was carried out using CASA, as described above [2].

### 2.3. Postharvest Processing of Mouse Testicular Tissues

Testes and epididymides from the mature mice were harvested, as detailed above. Unless indicated otherwise, all procedures were done at RT. Tissues were fixed in Bouin's solution for 48 h, washed in 70% ethanol (ETOH) until the yellow color of Bouin's disappeared (~48 hr with frequent changes of 70% ETOH and gentle agitation), and divided into four groups:* Control group*: tissue was stored in 70% ETOH until paraffin embedding;* Rapid transition*: 70% ETOH was immediately replaced with PBS (Sigma Aldrich, St. Louis, MO), pH 7.4 for one wk, and then rapidly replaced with 70% ETOH;* Slow Re-ETOH only*: 70% ETOH was immediately replaced with PBS for one wk and then sequentially replaced at 2 hr intervals with each of 10%, 30%, 50%, and 70% ETOH;* Slow rehydration-dehydration*: 70% ETOH was sequentially (70%, 50%, 30%, 10%, then PBS, at 2 hr each) substituted with PBS for one wk and then replaced at 2 hr intervals with each of 10%, 30%, 50%, and 70% ETOH. To prevent contamination, we kept tissue in PBS at 4°C, and ETOH replacement was completed at RT with 2 hr intervals between changes, as detailed above. After each of the respective final dehydration steps above, the tissues were paraffin-embedded and processed for histology and hematoxylin and eosin staining (HE) using standard methods as performed previously [3].

### 2.4. Total RNA Extraction and Preservation

Freshly harvested mouse testes were immediately stabilized in 10 volumes of TRIzol reagent (Invitrogen, Carlsbad, CA) or RNA*later* solution (Ambion, Austin, TX). The samples placed in RNA*later* were stored for (1) two wks at 4°C; (2) one wk at RT and one wk at 4°C; or (3) two wks at RT. Before RNA extraction, tissues were retrieved from RNA*later* solution with sterile forceps and then submerged in TRIzol reagent. RNA was isolated with TRIzol reagent according to the manufacturer's instructions. Time of placement into RNA*later* was noted to determine if RNA quality was related to the duration of window from euthanasia to placement in RNA*later*. RNA integrity and quantity were determined using Agilent RNA kit and Agilent Bioanalyzer 2100 (Santa Clara, CA). One *μ*g of RNA was subjected to RT PCR with the primers specific for mouse GAPDH (Forward: 5′CCTTCATTGACCTCAACTAC; Reverse: 5′ATGACAAGCTTCCCATTCTC), and interleukin-1alpha (IL-1*α*) (Forward: 5′ACTTGTTTGAAGACCTAAAG; Reverse: 5′GTTTCAGAGGTTCTCAGAG). Primers were designed using online Primer Design Tool Primer 3. Uteri and ovaries from STS-135 were harvested in RNA*later* solution and stored at −80°C for 10 months. Total RNA was isolated from ovaries and uterine horns with Gene Elute Mammalian RNA kit (Sigma Aldrich, St. Louis, MO). PCR products were verified on 3.0% agarose gels using standard procedures.

### 2.5. Protein Extraction from Ovaries and Uterus Tissue Preserved in RNA*later*

Ovaries stabilized in RNA*later* for one wk at RT and two wks at 4°C were used for protein extraction. The tissue was removed from RNA*later*, briefly rinsed with ice-cold PBS, pH 7.4, and then homogenized in RIPA buffer containing protease inhibitor cocktail (all from Sigma Aldrich, St. Louis, MO) or ProteoJet Mammalian Cell Lysis Reagent (Fermentas, Pittsburgh, PA). Uteri from STS-135 mission mice were preserved in RNA*later* for 10 months at −80°C, prior to processing in RIPA as described above. Protein concentration was determined using the DC Assay (Bio-Rad, Hercules, CA), and 15 *μ*g protein was electrophoresed under denaturing conditions on 4–15% polyacrylamide gel and transferred to nitrocellulose membrane (Bio-Rad, Hercules, CA). The membranes were blocked for 1 hr with 5% nonfat milk in TBS-T (Tris buffered saline with Tween-20, Sigma Aldrich, St Louis, MO) and probed with 1 *μ*g/mL rabbit anti-mouse estrogen receptor alpha (ER*α*) antibody (Santa Cruz Biotechnology, CA). To verify equal loading of proteins, membranes were stripped and reprobed with a goat anti-*β*-actin antibody (Santa Cruz Biotechnology, CA). Incubations with primary antibodies were carried out overnight at 4°C. After washing in TBS-T and probing with the corresponding horseradish peroxidase-labeled secondary antibody (Pierce Biotechnology, Rockford, IL), bound antibodies were identified using Amersham ACL Plus Western Blotting Detection Reagents (GE Healthcare, Pittsburgh, PA) and luminography. Western blots were quantitated by densitometric analysis.

## 3. Results

### 3.1. Effect of Delayed Processing on Quality of Mouse and Gerbil Reproductive Tissue Histology

#### 3.1.1. Male Mouse

**Figure 1:**
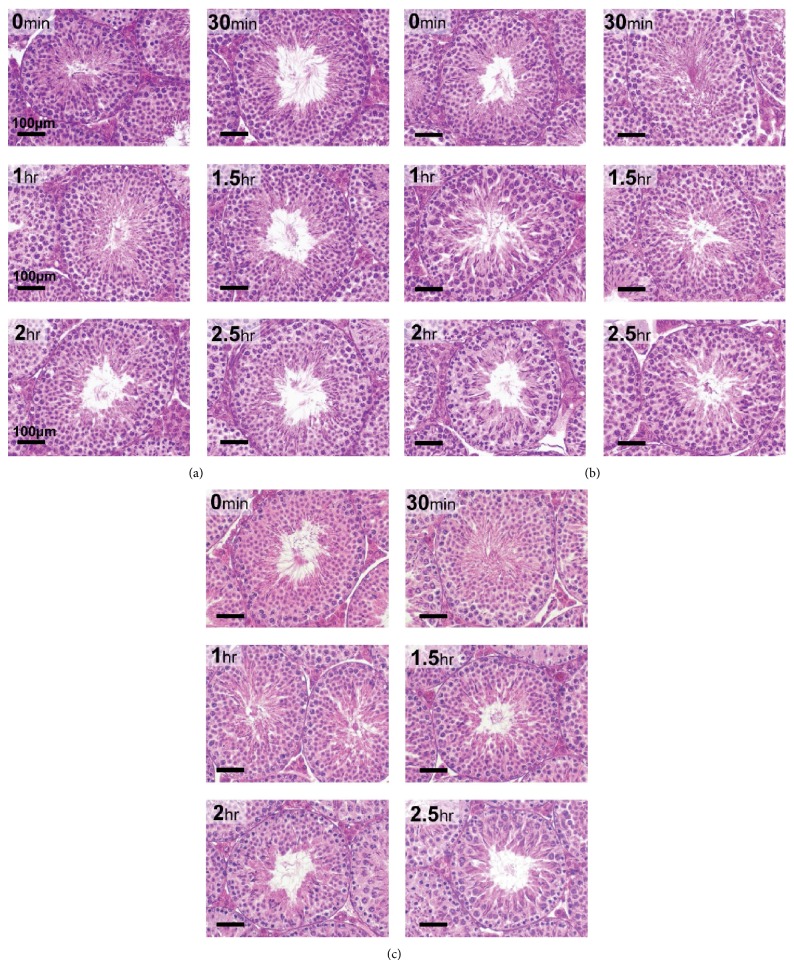
Effect of delayed processing on C57Bl/6J testicular morphology. Each panel represents light microscopy (40x objective) of sections of adult mouse testis stained with hematoxylin and eosin (HE) (magnification bar is 100 *μ*m). (a) Tissues kept on ice for 0 to 2.5 hr after harvesting; (b) tissues allowed to remain in the carcass for 0 to 2.5 hr; (c) tissues removed and kept at RT for 0 to 2.5 hr in Ham's F10 after harvesting.

*Testes* harvested any time between time 0 after euthanasia up to 2.5 hr after euthanasia showed histological properties comparable to the time 0-harvested mice. For comparative purposes, the 0 min harvested testes represent the control for subsequent time points for each storage treatment, respectively. Histological quality of the testes was excellent in all postharvest treatment procedures; namely, tissues were kept in the carcass until fixation or fixed in Bouin's immediately or stored in Hams F10 on ice or RT before fixation. Testis tubules appeared normal in all treatment groups compared to the 0 min controls, with no shrinkage of tissue and complete retention of histologic architectural details (Figures 1(a), 1(b), and 1(c)).

*Total motility and progressive motility of cauda epididymal mouse sperm at times* 0, 0.5, 1.0, 1.5, 2.0, and 2.5 hr were not significantly different between any of the tissue harvest time points and also not significantly different between the three tissue harvest scenarios.  Table 1 shows the percent progressive motility for each time point under all three tissue storage conditions.  Table 2 shows the percent total motility for each time point under all three tissue storage conditions. Based on comparable results between the three tissue harvest regimens, we focused on the “tissue in carcass” at RT regimen for subsequent experiments with female mice, as well as male and female gerbils.

#### 3.1.2. Male Gerbil

**Figure 2:**
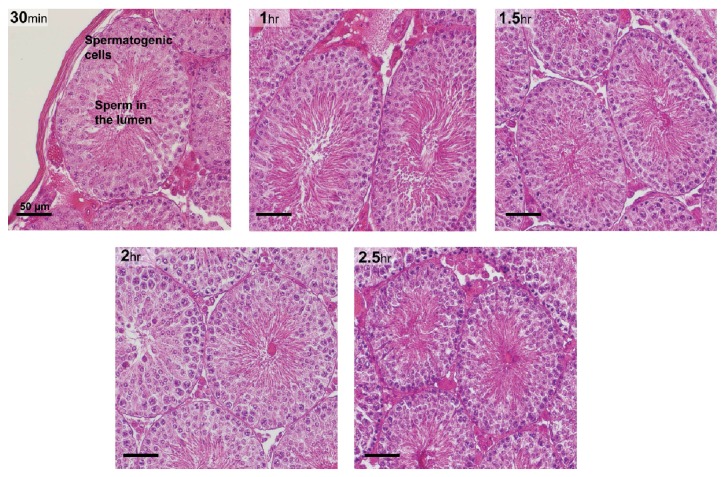
Gerbil testicular morphology (40x objective) at various time points after tissue harvest from the carcass (magnification bar is 50 *μ*m). All gerbils were euthanized at once and tissues were harvested from the carcass at 0.5 hr interval from 0 to 2.5 hr. HE staining demonstrated the retention of histological features at every time point.

*Testis* showed normal distinct histological details with all spermatogenic cells arranged in a normal pattern in the tubule when collected up to 2.5 hr after euthanasia (Figure 2). Total motility of the gerbil cauda epididymal sperm harvested at each at time point was analyzed and is presented in Table 3. Since the data represent a single animal, statistical analysis cannot be done. Given the variation in motility with time, the data suggest that motility was relatively stable at all time points, except with perhaps a drop at 2.5 hr.

#### 3.1.3. Female Mouse

**Figure 3:**
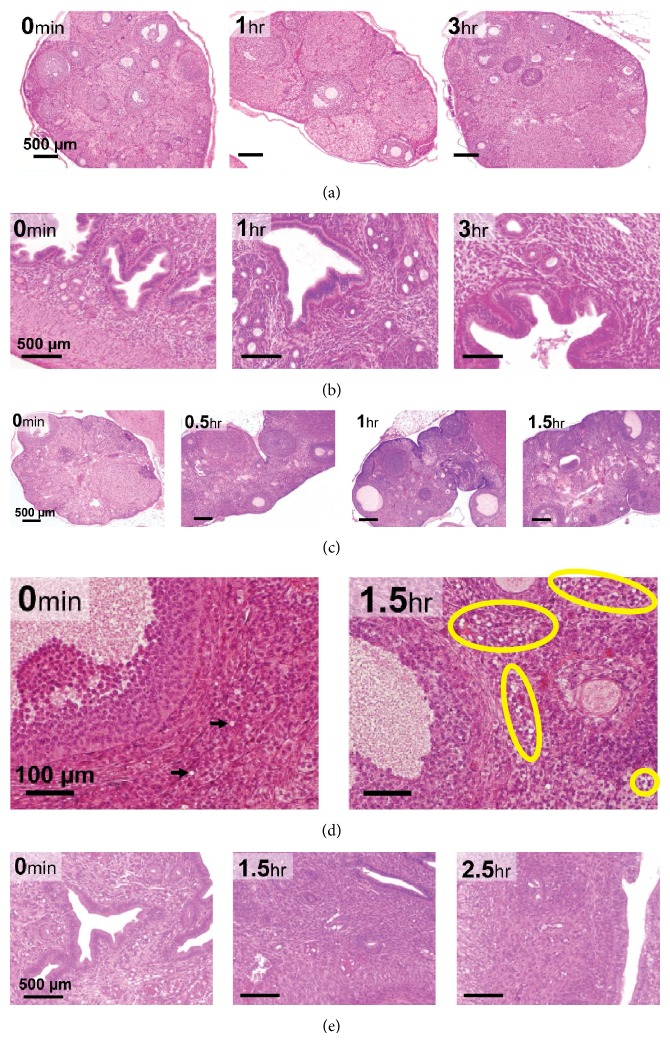
Mouse and gerbil ovarian and uterine horn histology. HE staining was used to evaluate quality of oocyte and follicles. Rows represent HE staining of: (a) mouse ovary up to 3 hr after euthanasia (4x objective, magnification bar is 500 *μ*m); (b) mouse uteri up to 3 hr after euthanasia (10x objective, magnification bar is 500 *μ*m); (c) gerbil ovary up to 1.5 hr after euthanasia (4x objective; magnification bar is 500 *μ*m); (d) significantly high number of vacuoles are indicated in the yellow circles in gerbil ovaries from 1.5 h after euthanasia (40x objective, magnification bar is 100 *μ*m); (e) gerbil uteri up to 2.5 hr after euthanasia (10x objective, magnification bar is 500 *μ*m).

The* ovaries (Figure 3(a)) and uteri (Figure 3(b))* harvested from female mice up to 3 hr after euthanasia showed excellent histological properties devoid of apparent tissue degradation.

#### 3.1.4. Female Gerbil

Gerbil* ovaries* (Figure 3(c)) harvested up to 1 hr after euthanasia showed normal healthy follicles and healthy oocytes devoid of signs of tissue degradation; however at 1.5 hr after euthanasia high numbers of unhealthy follicles and shrunken oocytes were seen, suggesting tissue deterioration due to the delay in fixation process after euthanasia. Significantly high numbers of “vacuoles-like” structures were also seen at 1.5 hr after euthanasia (Figure 3(d)), which is indicative of tissue degradation (indicated by the yellow circles). Control (0 min) gerbil ovaries had negligible “vacuoles” (indicated by two black arrows). The ovary harvested after 1.5 hr did not sustain the histological processing as they were too fragile and degraded, implying that gerbil ovaries were more sensitive and should be harvested within 1 hr of euthanasia.* Uterine* histology indicated tissues were intact and comparable to control at all tested time points (Figure 3(e)).

### 3.2. Effect of Different Postfixation Procedures on Quality of Testicular Histology

**Figure 4:**
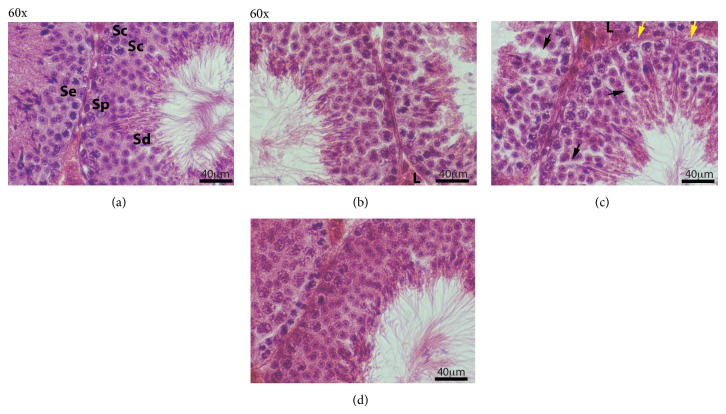
Morphological analysis of mouse testis after different postfixative manipulations. Sections of adult mouse testis were stained with HE (all at 60x; magnification bar is 40 *μ*m). (a) Control; (b) slow rehydration-dehydration stepwise replacement of ETOH-PBS-ETOH; (c) slow Re-ETOH* only* stepwise replacement; (d) rapid ETOH-PBS-ETOH transition. Sertoli cell (Se), spermatogonia (Sp), spermatocytes (Sc), spermatids (Sd), and Leydig cell (L).* Black arrows*—abnormal open spaces in seminiferous epithelium;* yellow arrows*—abnormal wavy and thinner basement membrane.

**Figure 5:**
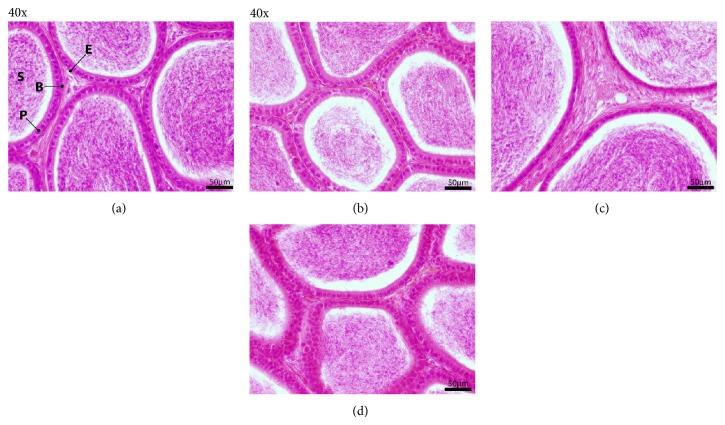
Morphological analysis of mouse epididymis after different postfixative modifications. Sections of adult mouse epididymis were stained with HE (all at 40x; magnification bar is 50 *μ*m). (a) Control; (b) slow rehydration-dehydration stepwise replacement of ETOH-PBS-ETOH; (c) slow Re-ETOH* only* replacement; (d) rapid ETOH-PBS-ETOH transition. Epithelium (E), sperm (S), basal cell (B), and principal cell (P).

Testicular and epididymis morphology was evaluated for histological changes after different postfixation processing (Figures 4 and 5). In control testicular tissue (Figure 4(a)), all types of spermatogenic cells, spermatogonia (Sp), Sertoli cells (Se), spermatocytes (Sc), and spermatids (Sd), were evident. Lymphatic spaces between seminiferous tubules and adjacent to Leydig cells (L) clusters are clearly defined. After the slow rehydration-dehydration stepwise replacement with ETOH, testis tubules appeared normal with histological architecture similar to the control group (Figure 4(b)). Although all types of spermatogenic cell were identified after slow rehydration-dehydration stepwise replacement, spermatogonia and Sertoli cell nuclei were more difficult to distinguish compared to control. The quality of testicular histology observed after slow Re-ETOH only (Figure 4(c)) was similar to that observed in the slow rehydration-dehydration stepwise replacement group. However, open spaces (black arrows) were observed within portions of seminiferous tubules in the slow Re-ETOH only group, and the seminiferous tubule basement membrane in this group often appeared wavy and thinner (yellow arrows) compared to the control (Figure 4(a)). The histologic quality was poor in the group treated by single step (rapid transition) change of solution (Figure 4(d)), as evidenced by a diffuse appearance of the tissue (not due to focus) and limited clarity of nuclear details in spermatogonia and Sertoli cells. In addition, the seminiferous tubule basement membrane was occasionally indistinct and some spermatid artifactual loss of residual bodies is also observed.

We found epididymal tissue to be sensitive to dehydration/rehydration shock (Figure 5). Slow rehydration-dehydration ETOH replacement had no visible negative effect on the quality of epididymal morphology (Figure 5(b)); however histological examination revealed differences in epididymal morphology after slow Re-ETOH only (Figure 5(c)) and rapid transition procedures (Figure 5(d)), compared to control (Figure 5(a)). The differences included alterations in the thickness of columnar epithelium, and basal and principal cells are not very sharp. The* slow rehydration-dehydration* caused less destruction of testicular and epididymal tissue than the rapid single-step changes of solution and has an overall better morphological detail preservation compared to the rapid ETOH-PBS solution change.

### 3.3. Total RNA and Protein Evaluation after Tissue Preservation in RNA*later* under Long-Term Storage

#### 3.3.1. RNA Stability

**Figure 6:**
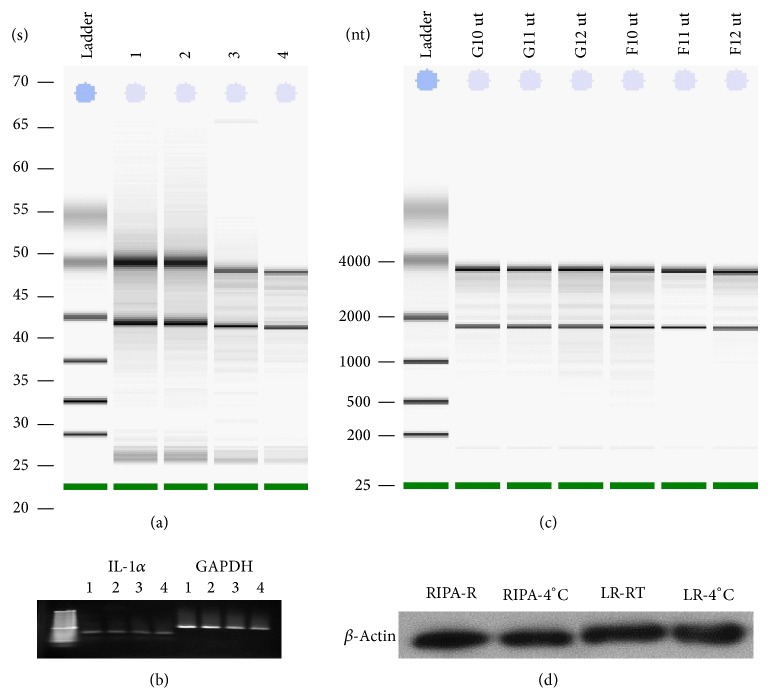
Effect of different extraction and storage methods on RNA and protein quality in ovaries, and testes extracted and/or stabilized in TRIzol or RNA*later*. (a) Total RNA integrity analysis. Total RNA was isolated from mouse testis and stabilized in TRIzol reagent (RNA yield and quality control (lane 1); RNA*later* for 2 wks at 4°C (lane 2); RNA*later* for 1 wk at room temperature and 1 wk at 4°C (lane 3); RNA*later* for 2 wks at RT (lane 4). (b) Agarose gel electrophoresis of PCR products with primers for IL-1*α* and GAPDH. For RT PCR total RNA was used after stabilization in TRIzol reagent (lane 1); RNA*later* for 2 wks at 4°C (lane 2); RNA*later* for 1 wk at RT and 1 wk at 4°C (lane 3); RNA*later* for 2 wks at RT (lane 4). (c) Total RNA integrity analysis STS-131 ground (G10, G11, and G12) and flight (F10, F11, and F12) uteri fixed in RNA*later* after 30–40 min after euthanasia. (d) Comparison of buffers to remove RNA*later* for subsequent western analysis of *β*-actin integrity in ovaries. Mouse ovaries were stored at RNA*later* for 1 wk at RT or 2 wk at 4°C; tissues were homogenized in RIPA lysis buffer (lanes 1, 2) or ProteoJET Lysis reagent (LR) (lanes 3, 4). Total cell lysates were prepared and subjected to SDS-PAGE. Western for *β*-actin is presented.

We compared RNA integrity after preservation testicular tissue in TRIzol reagent and RNA*later* (Figure 6(a)). Total RNA integrity analysis demonstrated high quality RNA after storing tissue in RNA*later* for 1-2 wk at RT or 4°C compared to TRIzol preservation. Distinct 28S and 18S ribosomal RNA and absence of degraded RNA were observed on the gel. RT-PCR analysis of RNA with primers for GAPDH and IL-1*α* indicated high quality of expected PCR products in all analyzed samples (Figure 6(b)). Integrity analysis of total RNA isolated from STS-131 ground controls (G10, G11, and G12) and flight (F10, F11, and F12) mouse uteri after 30 to 40 min after euthanasia demonstrate high RNA quality in these samples (Figure 6(c)). We found no differences in RNA quality between all analyzed uteri and ovaries samples placed into RNA*later* from 15 min to 40 min after euthanasia.

#### 3.3.2. Long-Term Storage for RNA Analysis

**Figure 7:**
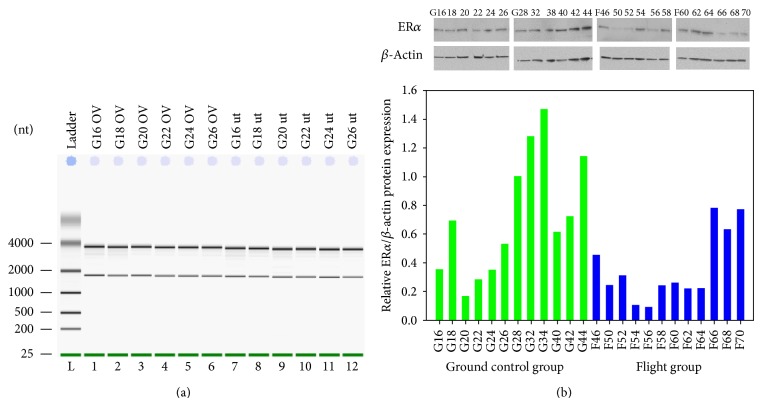
RNA and protein quality of STS-135 uteri and ovaries stabilized in RNA*later* for 10 months at −80°C. (a) STS-135 ground control (G16–G26) ovarian and uterine RNA integrity analysis. Total RNA was extracted and examined for RNA quality; (b) Western blot analysis of ER*α* and actin in STS-135 mouse uterus. Total cell lysates were prepared and subjected to SDS-PAGE (15 *μ*g/lane). Western blot analysis was performed using the corresponding antibodies to check expression levels of the proteins. Representative Immunoblot (top) and its graphical presentation (bottom). Densitometric intensities of specific protein bands were digitally obtained and normalized to *β*-actin.

Using ovary and uteri harvested from STS 135 mice, we also determined the effect of long-term (10 months) preservation in RNA*later* on RNA and protein quality (Figure 7). This time frame was chosen to mimic a possible storage scenario that could occur on the ISS. Ovaries and uterus from STS-135 ground controls stabilized in RNA*later* for 10 months showed excellent RNA quality (Figure 7(a)) and yield in range 6–8 *μ*g.

#### 3.3.3. Protein Stability

Although not commonly done, tissue stabilized in RNA*later* can be used for subsequent protein extraction. Protein obtained from samples stored in RNA*later* is suitable for western blotting or 2D gel electrophoresis, but not for applications that require native protein (Ambion Guideline for RNA*later*). We optimized the protocol for protein extraction from ovaries preserved in RNA*later* for one wk at RT or two wks at 4°C by using RIPA buffer or ProteoJet Lysis reagent (LR). Western analysis of *β*-actin integrity demonstrated that one wk ambient storage of testicular tissue in RNA*later* did not affect actin integrity as evidenced by the absence of proteolytic fragments and consistent signal intensity in replicate samples (Figure 6(d)).

#### 3.3.4. Long-Term Storage for Protein Analysis

Protein from uteri stabilized in RNA*later* for 10 months after STS-135 mission was extracted with RIPA buffer. Immunoblot for ER*α* and *β*-actin verified excellent expression levels of both proteins with no evidence of proteolytic degradation of both proteins (Figure 7(b)). Quantitative densitometry analysis of western blots indicated reduced levels ER*α* in mostly flight animals. These results demonstrate that RNA*later* is an effective sample collection and stabilization reagent for protecting both RNA and protein under long-term conditions compatible for the ISS.

## 4. Discussion

We report here a logical method to determine the optimal time window of tissue harvest and fixation after euthanasia for use in multi-investigator tissue harvest programs that is compatible with processing of tissue sample obtained from space flight animals. We also demonstrate here a long-term storage regimen for animal tissues compatible with recovery of high quality RNA and protein under conditions similar to that on the ISS, when there may be many months between sample collection and return to Earth. The procedures set forth also include methods for tissue harvest at the site of return and for safe shipment to external laboratories for further processing for histopathology and recovery of protein and RNA. Several fixation/preservation studies have been carried out for plant samples [1, 4, 5]. One European Space Agency report included a brief discussion on fixation of mammalian cells in tissue culture for microscopy [6]. Freidin et al. [7] have demonstrated significant alterations on gene expression in lung carcinoma tissues collected about 30 minutes after harvest. Durrenberger et al. [8] reported that, in human brain samples collected from several brain banks, antemortem events appeared to negatively affect the RNA quality, but postmortem delays caused no significant deterioration. This observation supported earlier report that postmortem delay had negligible effect on RNA quality [9]. Human stomach has been described as the tissue showing the earliest sign of postmortem [10]. Presnell and Cina described stomach and pancreas as the earliest human tissues to deteriorate following death [11]. The significance of quick processing of histopathological specimen in a clinical setting has been identified by Rohr et al. [12]. However, we have not come across similar studies for animal tissues used in biomedical research. Prior to our study reported here, there was a significant knowledge gap in the literature for methods to process animal tissues compatible for multi-investigator Biospecimen Sharing Programs for space flight logistical scenarios. For our flight studies, using male and female mice on three different space shuttle flights and the BION M1 flight, it was critical to determine the optimum conditions of tissue harvest and processing for tissues of our interest, namely, testis, epididymis, ovary, and uteri.

Space flight studies usually comprise remotely dispersed multi-investigator collaborations. Thus, there is a need to ship tissue samples from the site of collection at the return-to-Earth laboratory facility for initial tissue harvest to the site of final processing and detailed data collection. With respect to collection of RNA samples, TRIzol reagent gives excellent RNA integrity; however it is a phenol-based solution and tissue preserved in TRIzol cannot be shipped internationally due to airline safety restrictions. Lyophilization of fresh tissue specimen has been shown to preserve RNA and protein quality and levels by Wu et al. [13]. Though lyophilization makes shipping easier, especially across international borders, it is not a viable option for preservation of highest quality histological analysis. To obtain optimum RNA and protein quality, our results demonstrate that mouse testicular tissue can be submerged in RNA*later* and stored successfully for analysis at a later time point, at least 10 months at −80°C. It should be noted that 10 months represents a minimum limit, and longer storage intervals would still need to be directly assessed. Given the current estimate for ISS SpaceX Dragon flights at approximately 3-month intervals, this would span three opportunities for sample return after an experiment is terminated and ensure maintenance of RNA and protein sample quality. It is known that, in order to isolate high quality RNA and protein from mammalian tissue, the tissue must be processed directly after harvest. We determined, for our tissues of interest, that excellent RNA stability was achieved if the tissue samples were placed into RNA*later* up to 40 min after euthanasia. RNA*later* is a popular reagent that inactivates all cellular enzymes, including RNAses; thus RNA expression profiles can be preserved in situations when immediate RNA isolation is not feasible. The tissue can be stored in RNA*later* for a long time without nucleic acid degradation. RNA*later* has been used by investigators for collection of human tissue [14] and used in RNA expression microarrays [15]. Our results indicated that RNA*later* enables long-term tissue preservation for RNA and protein extraction compatible with delayed sample return from flight. This process should be evaluated for use on other tissues to maximize optimal histology and gene transcription data collection in the primary flight experiments as well as the Biospecimen Sharing Programs (BSP) investigators. Freidin et al. [7] have confirmed the significance of RNA*later* as a medium to preserve gene expression of lung tissues. Tissue processing methods should be standardized for best storage and analysis of harvested tissues. Our methods for tissue fixation, long-term storage, and recovery of protein and RNA are compatible for planned in-flight tissue harvest on ISS.

With regard to obtaining the best possible tissue fixation for histology collected under a multi-investigator Biospecimen Sharing Program, obtaining many tissues of interest to the participating investigators has to be considered in an integrated way to accommodate the scientific requirements of the overarching flight project, as well as the natural degradation process that tissues undergo as soon as euthanasia has occurred. Depending on the tissue and species, different windows of time from euthanasia to fixation may exist within which histological architecture (as well as RNA and protein integrity) is stable. In the study design reported here, we have determined the window of time and temperature for optimal postharvest maintenance of male and female tissue quality (testis, epididymis, ovary, and uterine horn) and sperm viability in mice and gerbils. These harvest protocols provide a logical method for integrating the tissue flow logistics for postflight animals for any project involving multiple investigators. Protocols compatible with investigators who require more rapid tissue retrieval can be identified and prioritized to ensure data preservation. We determined that mouse testes were able to retain excellent histological details when processed up to 3 hr after euthanasia. Sperm motility showed gradual decline with time. The authors would reiterate as mentioned in the Results section that sperm motility is a highly sensitive and variable parameter. It is normal to see major differences in motility of sperm obtained from not only one mouse to another, but also within samples obtained from the same mouse or gerbil. Nonetheless, even within the span of variability seen, our results indicate that, in case of mouse, there was no major difference in sperm motility between any of the time points, whereas in case of gerbil, we saw suggestions of a slight drop at 2.5 hr after harvest. Future studies for flight will require analysis of larger *n*'s during definition phase, if gerbils will be used. Of the tissues studied here, Mongolian gerbil ovaries appear most sensitive to delay in processing, and require more rapid posteuthanasia processing than mouse ovaries. Determining optimum conditions for tissue handling after harvest is very crucial and can help in maximizing tissue retrieval form animal models thereby maximizing data output. Based on the time-sensitivity, investigators may be able to plan the sequence in which the tissues are harvested starting with the most sensitive tissue to least sensitive.

Finally, with regard to the requirement to ship tissues fixed for histopathology, airline and ground transport providers (especially international carriers) have specific safety regulations that prohibit shipment of samples containing many widely used fixatives and preservatives of tissue histologic integrity. Science requirements may present challenges in using alternative fixatives. In this regard, Bouin's fixative (which contains picric acid) is the best fixative for the testis if the tissue is to be embedded in paraffin [16]. However, in space flight experiments, safety regulations prevent its use on flight platforms and its presence in tissue samples being shipped. Thus, we optimized protocols for fixative removal and storage of tissues in PBS for safe shipping, as well as reconstitution protocols for storing tissues in 70% ethanol to retain excellent histology.

## 5. Conclusion

Optimal time frames for harvesting testis, epididymis, ovary, and uteri without compromising the histological quality, sperm motility, and RNA quality have been determined. Differences in tissue-specific optimal fixation time windows were noted between mice and gerbils. We provide here new methods for (1) fixative removal and transfer of tissues into aqueous media for safe shipping and (2) reconstitution protocols into 70% ethanol that retains excellent histology. We conclude that stepwise replacement of ETOH-PBS-ETOH caused less degradation of histological quality of tissue than a single-step change of solution. Our results demonstrate that male and female mouse reproductive tissues stored in RNA*later* solution were stable and gave high quality RNA and protein after 10 months of storage at −80°C. Thus, we have determined methods for postharvest tissue processing to replace Bouin's with 70% ethanol for safe shipping across USA and also replace 70% ethanol with PBS to enable shipping of tissues across international borders. These protocols will facilitate integration of tissue harvest logistics in multi-investigator Biospecimen Sharing Programs for optimal tissue histology and retention of high quality RNA and protein recovery from animal tissues on long-term space flight experiments on ISS as well as other flight platforms.

## Figures and Tables

**Table 1:** Percent progressive motility (±SD) of mouse cauda epididymal sperm after exposure of epididymis to various conditions.

Time after euthanasia	In carcass	On ice^*^	^*^At room temperature
0 h	23 ± 18	25 ± 26	26 ± 19
0.5 h	28 ± 13	22 ± 13	27 ± 12
1.0 h	31 ± 3	32 ± 19	31 ± 9
1.5 h	27 ± 6	47 ± 9	30 ± 11
2.0 h	26 ± 7	18 ± 9	28 ± 14
2.5 h	26 ± 9	23 ± 12	25 ± 16

^*^Tissue was submerged in Ham's F-10 medium in a 15 mL tissue culture tube which was placed on ice or at room temperature. Values are mean ± standard deviation (*n* = 2 mice at each time point). There was no significant difference in motility between the three testing conditions at each time point.

**Table 2:** Percent total motility (±SD) of mouse cauda epididymal sperm after exposure of epididymis to various posteuthanasia conditions.

Time after euthanasia	In carcass	On ice^*^	^*^At room temperature
0 h	48 ± 18	49 ± 29	45 ± 21
0.5 h	51 ± 12	47 ± 11	50 ± 15
1.0 h	53 ± 6	60 ± 21	40 ± 9
1.5 h	57 ± 12	67 ± 16	54 ± 11
2.0 h	51 ± 14	46 ± 14	43 ± 14
2.5 h	50 ± 17	49 ± 18	52 ± 12

^*^Tissue was submerged in Ham's F-10 medium in a 15 mL culture tube which was placed on ice or at room temperature as indicated. Values are mean ± standard deviation (*n* = 2 mice at each time point). There was no significant difference in motility between the three testing conditions at each time point.

**Table 3:** Percent total motility of gerbil cauda epididymal sperm recovered from the epididymis after storage in the carcass at RT for the times indicated (*n* = 1 at each time point).

Time after euthanasia	Total motility (%)	Progressive motility (%)
0 h	98.3	85.0
0.5 h	82.4	58.0
1.0 h	82.6	61.0
1.5 h	95.2	86.2
2.0 h	87.9	73.2
2.5 h	75.6	47.5

Since we used one animal per time point, standard deviation could not be determined.

## Abbreviations

TBS-T: Tris buffered saline with 0.1% Tween 20
BSP: Biospecimen Sharing Program
ETOH: Ethyl alcohol
STS: Space Transport System
PBS: Phosphate buffered saline
ISS: International Space Station
CASA: Computer assisted sperm analysis
Wk: Week
RT: Room temperature
IACUC: Institutional Animal Care and Use Committee
KSC: Kennedy Space Center
RNA: Ribonucleic acid
CO_2_: Carbon dioxide.
